# Predictors of renal function deterioration at one year after off-clamp non-renorrhaphy partial nephrectomy

**DOI:** 10.1371/journal.pone.0303104

**Published:** 2024-05-13

**Authors:** Masaki Nakamura, Shuji Kameyama, Ibuki Tsuru, Taro Izumi, Akihiro Ono, Taro Teshima, Yasushi Inoue, Ryo Amakawa, Hiroki Inatsu, Tadashi Yoshimatsu, Masashi Kusakabe, Teppei Morikawa, Yoshiyuki Shiga

**Affiliations:** 1 Department of Urology, NTT Medical Center Tokyo, Tokyo, Japan; 2 Department of Radiology, NTT Medical Center Tokyo, Tokyo, Japan; 3 Department of Diagnostic Pathology, NTT Medical Center Tokyo, Tokyo, Japan; Tanta University Faculty of Medicine, EGYPT

## Abstract

**Background:**

Preservation of renal function is an important goal in renal cell carcinoma-related surgery. Although several case-dependent techniques for renal pedicle clamping and hemostasis have been used, their effects on long-term renal function are controversial.

**Methods:**

The clinical records of 114 patients who underwent off-clamp non-renorrhaphy open partial nephrectomy at our hospital were retrospectively reviewed. Perioperative estimated glomerular filtration rate (eGFR) preservation was calculated, and predictors of eGFR decline 12 months post-surgery and overtime deterioration of renal function were identified using a multivariate regression analysis.

**Results:**

The median patient age was 65 years, and the median tumor size was 27 mm. The mean eGFR preservation at 1, 3, and 12 months post-surgery were 90.1%, 89.0%, and 86.9%, respectively. eGFR decline at 1 and 3 months were associated with poor eGFR preservation at 12 months with the odds ratio (95% confidence interval (CI)) of 1.97 and 3.157, respectively. Multivariate regression analyses revealed that tumor size was an independent predictor of eGFR decline at 12 months. Among 65 patients with eGFR preservation over 90% at 1 month post-surgery, eGFR value of 28 patients deteriorated below 90% at 12 months post-surgery compared with preoperative eGFR. Tumor size and eGFR preservation at 1 month were independent predictors of long-term renal function deterioration.

**Conclusion:**

Tumor size predicted eGFR decline 12 months post-surgery. Only a mild decline in eGFR was observed between 3 and 12 months after open partial nephrectomy. Tumor size and eGFR preservation at 1 month predicted the deterioration of renal function over time.

## Introduction

As the preservation of renal function is one of the important goals in surgery for RCC, partial nephrectomy (PN) has become the gold standard for renal tumors less than 7 cm in diameter and the means of hemostasis after tumor resection vary among institutes [1–3]. Although cortical renorrhaphy and soft coagulation are the most popular methods of hemostasis, the effect of each method on long-term renal function remains a subject of research [4, 5].

Introduced in PN, renorrhaphy aims to achieve hemostasis and close the urinary collecting system, thereby minimize postoperative complications. However, the pros and cons of renorrhaphy in terms of preserving renal parenchima have been widely debated [4, 6–8]. Renorrhaphy carries the risk of damaging adjacent renal vessels, potentially leading to reduced functioning renal parenchyma after surgery. Additionally, it may contribute to the development of renal artery aneurysms [9].

Techniques such as selective or super-selective clamping at the renal hilum can reduce renal parenchymal ischemia and better preservation of postoperative renal function [10, 11]. However, parenchymal ischemia and reperfusion resulting from hilar clamping could lead to acute kidney injury through production of radical oxygen species. Omitting both renal hilar clamping and renorrhaphy has been reported as an effective approach for preserving renal function during partial nephrectomy in a relatively short period [2, 12–15].

We have been performing off-clamp and non-renorrhaphy open partial nephrectomies for years. While most partial nephrectomies are currently performed laparoscopically or robotically, valuable lessons from open partial nephrectomy can be extrapolated to robotic procedures. In this study, we assessed renal function at 1 year after open off-clamp and non-renorrhaphy partial nephrectomies and investigated the predictors of renal function decline over time.

## Materials and methods

### Patients

Among 220 patients with renal tumors who underwent off-clamp non-renorrhaphy open partial nephrectomy at our institute between 2013 and 2020, those without follow-up until 12 months after surgery were excluded. Finally, the clinical records of the 114 patients were retrospectively analyzed. The data were accessed for research purpose on 1^st^ December, 2022. The authors did not have access to information that could identify individual participants during or after data collection.

### Assessment of renal tumor complexity

The complexity of renal tumors was analyzed according to the R-E-N-A-L nephrometry scoring system. Briefly, (R)adius (tumor size as maximal diameter), (E)xophytic and endophytic properties, (N)earness of the tumor to the collecting system or sinus, and (L)ocation relative to the upper and lower polar lines were scored on a 1-, 2-, or 3-point scale. The RENAL score represents the sum of R, E, N, and L scores [16].

### Surgical techniques

**Fig 1:**
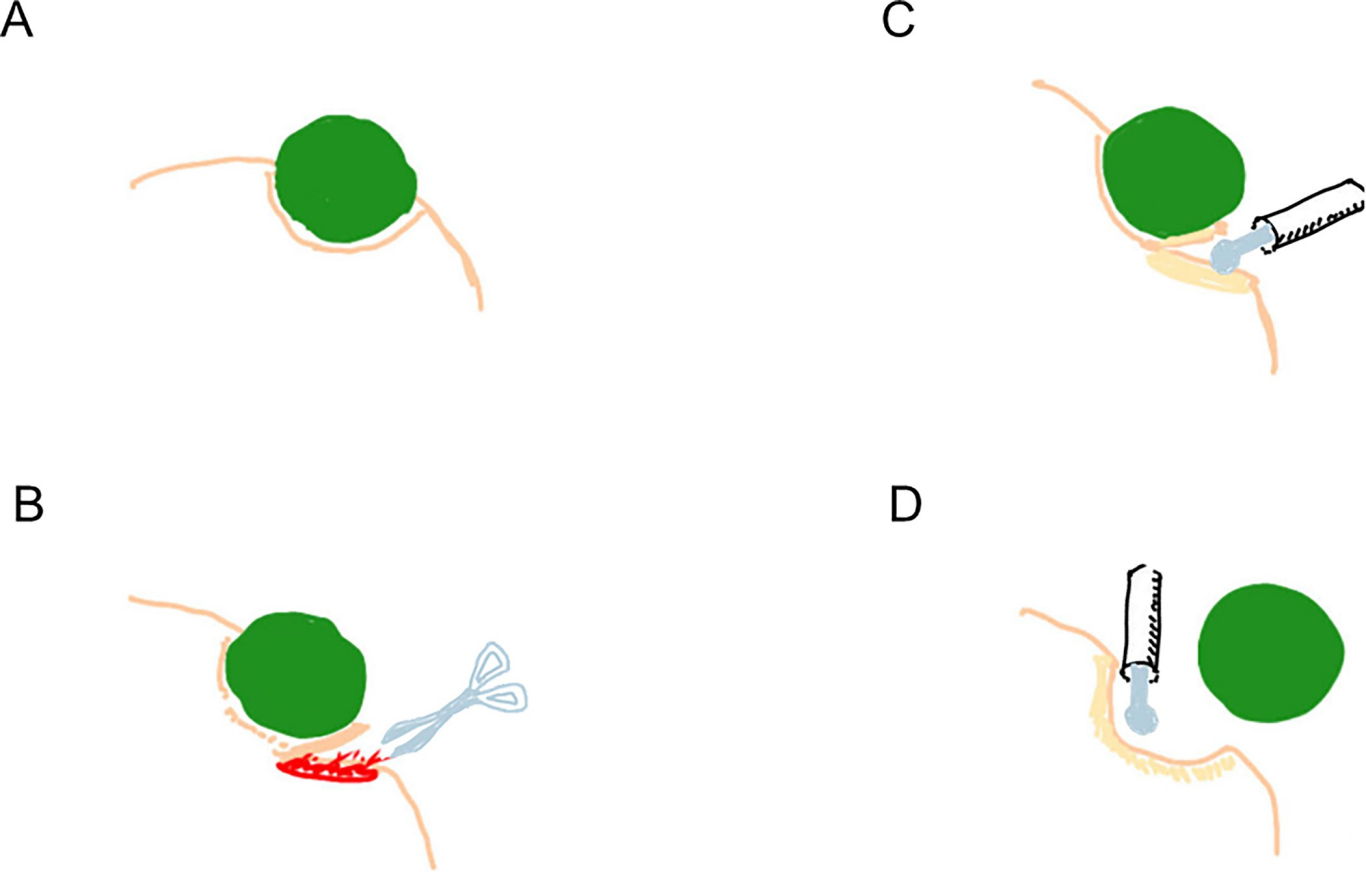
Surgical technique of partial nephrectomy. (A) Shema of a renal tumor. (B) Blunt separation and sharp cutting of the renal parenchyma with scissors. (C) Hemostasis using soft coagulation system. (D) Thorough hemostasis of the tumor bed with soft coagulation system.

All patients underwent retroperitoneal open partial nephrectomy as described previously [2]. Briefly, a partial nephrectomy was performed by blunt separation and sharp cutting, followed by hemostasis with monopolar SOFT COAG (VIO300D, ERBE, Germany). To minimize the blood loss, tumor resection was performed in millimeters. (Fig 1) The renal pedicle was not secured, and renal pedicle clamping and cortical renorrhaphy were omitted. The resection beds were sutured with 4–0 VICRYL^®^ only when the collecting system was opened. Urine leakage was ruled out by intravenous injection of an indigo carmine solution. TachoSil^®^ was placed on the resection surface to ensure hemostasis.

### Assessment of renal function and perioperative reduction in renal function

The estimated glomerular filtration rate (eGFR) was calculated using the equation 186 × (Creatinine/88.4)^-1.154^ × (age)^-0.203^ × (0.742, if female). The perioperative eGFR preservation at 5 days, 1 month, 3 months, and 12 months after surgery was calculated as postoperative eGFR/preoperative eGFR × 100 (%). According to the pentafecta definition after partial nephrectomy, we set the eGFR preservation threshold to 90% [3].

### Ethics approval and consent to participate

This study was approved by the Ethics Committee of NTT Medical Center, Tokyo (ID: 21–3).　Informed consent was obtained in the form of opt-out on the website. Those who were rejected were excluded. This study was conducted in accordance with the Declaration of Helsinki (revised in 2013).

### Statistical analysis

The chi-square test was used to analyze categorical variables. Univariate and multivariate regression analyses were performed to identify the predictors of postoperative eGFR preservation 12 months after surgery and overtime eGFR deterioration. The statistical significance was set at *p* < 0.05. All statistical analyses were performed using SPSS version 24 (IBM co. ltd., Tokyo, Japan).

## Results

　 The patient characteristics are described in Table 1. The median age and tumor size were 65 years (interquartile range (IQR), 54–69) and 27 mm (IQR, 19.5–40.5), respectively. Among the tumors, 35 tumors (30.7%) were >50% exophytic, 49 tumors (43.0%) were <50% exophytic, and 30 tumors (26.3%) were entirely endophytic. The median RENAL nephrometry score was 7 (IQR, 6–8). The surgical results are presented in Table 2. The median operative time and estimated blood loss were 124 min (IQR, 101–143) and 160 mL (IQR, 68–355), respectively. No major complications (Clavien–Dindo classification grade ≥ 3) occurred perioperatively. The mean eGFR value gradually decreased from 94.3% (standard deviation (SD), 14.0) at 5 days to 86.9% (SD, 12.9) at 12 months after operation.

**Table 1:** Patient and tumor characteristics.

No. patients	114
Sex (%)	
Male	80 (70.2)
Female	34 (29.8)
Median age (IQR)	65 (54–69)
Median BMI (IQR)	23. (21–26)
Median tumor size, mm (IQR)	27.0 (19.5–40.5)
Median RENAL nephrometry score (IQR)	7 (6–8)
Exophytic/Endophytic properties (%)	
> 50% exophytic	35 (30.7)
< 50% exophytic	49 (43.0)
Entirely endophytic	30 (26.3)
Location relative to the polar lines (%)	
Entirely above the upper or below the lower polar line	39 (34.2)
Lesion crosses polar line	56 (49.1)
> 50% of mass is across polar line or mass crosses the axial renal midline or mass is entirely between the polar lines	19 (16.7)
Comorbidity, n (%)	
Hypertension	43 (37.7)
Diabetes mellitus	16 (14.0)
Dyslipidemia	12 (10.5)

**Table 2:** Surgical results and complications.

Median operative time, min (IQR)	124 (101–143)
Median volume of estimated blood loss, mL (IQR)	160 (68.8 − 355)
No. additional resections	0
No. conversion to nephrectomy	0
Intraoperative blood transfusion	0
Postoperative blood transfusion	0
Pathology (%)	
Clear cell RCC	79 (69.3)
Papillary RCC	11 (9.6)
Chromophobe RCC	9 (7.9)
Angiomyolipoma	11 (9.6)
Others	4 (3.5)
Mean eGFR preservation, % (SD)	
Five days after surgery	94.3 (14.0)
One month after surgery	90.1 (11.5)
Three months after surgery	89.0 (13.7)
Twelve months after surgery	86.9 (12.9)

eGFR estimated glomerular filtration ratio, RCC renal cell carcinoma, IQR interquartile range, SD standard deviation

The tumor size and estimated blood loss were identified as predictors of eGFR preservation 12 months postoperatively. Multivariate analyses revealed only tumor size was an independent predictor of eGFR preservation at 12 months, with unstandardized regression coefficient B (95% confidence interval (CI)) of -0.183 (-0.316, -0.005) (*P* = 0007). (Table 3)

**Table 3:** Predictors of perioperative decline in eGFR at 12 months after surgery. Univariate and multivariate analysis.

	Univariate analysis			Multivariate analysis		
	Unstandardized regression coefficient B (95% CI)	Standardized regression coefficient β	*p -*value	Unstandardized regression coefficient B (95% CI)	Standardized regression coefficient β	*p -*value
Age^†^	-0.123 (-0.323, 0.077)	-0.114	0.225	-0.174 (-0.364, 0.016)	-0.162	0.072
BMI	-0.157 (-0.753, 0.439)	-0.05	0.602			
Tumour size^†^	-0.213 (-0.335, -0.091)	-0.31	0.01*	-0.183 (-0.316, -0.05)	-0.267	0.007*
Preoperative eGFR	-0.070 (-0.217, 0.077)	-0.089	0.345			
R	-6.827 (-11.068, -2.585)	-0.289	0.002*			
E	1.761 (-1.405, 4.927)	0.104	0.273			
N	-1.635 (-4.398, 1.128)	-0.11	0.224			
L	-2.073 (-5.522, 1.376)	-0.112	0.236			
RENAL score	-1.113 (-2.511, -0.284)	-0.148	0.117			
eBlood loss^†^	-0.014 (-0.023, -0.005)	-0.269	0.004*	-0.009 (-0.018, -0.001)	-0.165	0.091
HTN	-0.053 (-5.004, 4.898)	-0.002	0.983			
DM	-4.051 (-10.917, 2.816)	-0.11	0.245			
DL	-2.122 (-9.931, 5.687)	-0.051	0.591			

eGFR estimated glomerular filtration rate, BMI body mass index, eBlood loss estimated blood loss, R Radius, E exophytic and endophytic properties, N Nearness of the tumor to the collecting system or sinus, L location relative to the upper and lower polar lines, (R,E,N, and L were scored according to RENAL nephrometry scoring system), HTN hypertension, DM diabetes mellitus, DL dyslypidemia, CI confidence interval, ^†^ These factors were put in multivariate regression analysis, * p-value < 0.05 was considered statistically significant.

The eGFR preservation at 1 and 3 months was associated with poor eGFR preservation at 12 months. The calculated odds ratios (95%CI) were 1.97 (1.43, 2.71) and 3.157 (2.03, 4.90), respectively (Table 4). Among 65 patients with eGFR preservation over 90% at 1 month after surgery, the eGFR preservation of 28 patients deteriorated below 90% at 12 months after surgery. To further clarify the predictor of overtime eGFR deterioration from 1 month to 12 months, we performed univariate and multivariate analyses on patients with an eGFR preservation > 90% at one month after surgery. Tumor size and eGFR preservation at 1 month were the independent predictors of further deterioration in renal function, with unstandardized regression coefficients B (95% CI) of -0.189 (-0.378, -0.001) and 0.425 (0.096, 0.755), respectively (*P*-value, 0.048 and 0.012, respectively) (Table 5).

**Table 4:** The comparison of eGFR preservation ratio between 1, 3, and 12 months after operation.

	eGFR at 12 months ≥ 90%	eGFR at 12 months < 90%	OR	95% CI	*p value
eGFR at 1 month ≥ 90%	36	28	ref		
eGFR at 1 month < 90%	9	41	1.971	1.431–2.717	< 0.001
eGFR at 3 months ≥ 90%	35	17	ref		
eGFR at 3 months < 90%	10	52	3.157	2.031–4.908	< 0.001

CI confidence interval, eGFR estimated glomerular filtration ratio, OR odds ratio, * pearson’s chi-square test

**Table 5:** Predictors of overtime decline in eGFR (from 1 month to 12 months after surgery). Univariate and multivariate analysis.

	Univariate analysis			Multivariate analysis		
	Unstandardized regression coefficient B (95% CI)	Standardized regression coefficient β	*p -*value	Unstandardized regression coefficient B (95% CI)	Standardized regression coefficient β	*p -*value
Age^†^	-0. 006 (-0.171, 0.184)	0.009	0.945	-0.053(-0.233, 0.127)	-0.076	0.558
Tumour size^†^	-0.173 (-0.327, -0.02)	-0.276	0.027*	-0.189 (-0.378, -0.001)	-0.303	0.048*
Preoperative eGFR	0.052 (-0.106, 0.209)	0.083	0.514			
RENAL score	-0.793(-2.104, -0.518)	-0.152	0.231			
eBlood loss^†^	-0.005 (-0.016, -0.007)	-0.098	0.441	0.004 (-0.009, 0.017)	0.085	0.547
HTN	-0.053 (-5.004, 4.898)	-0.002	0.983			
eGFR preservation at one month^†^	0.482 (0.155, 0.808)	0.353	0.005*	0.425 (0.096, 0.755)	0.312	0.012*

eGFR estimated glomerular filtration rate, BMI body mass index, eBlood loss estimated blood loss, R Radius, E exophytic and endophytic properties, N Nearness of the tumor to the collecting system or sinus, L location relative to the upper and lower polar lines, (R,E,N, and L were scored according to RENAL nephrometry scoring system), HTN hypertension, CI confidence interval, ^†^ These factors were put in multivariate regression analysis, * p-value < 0.05 was considered statistically significant.

## Discussion

In our previous report, we examined perioperative renal function within 3 months after off-clamp, non-renorrhaphy open partial nephrectomy [12]. Notably, the renal function at 1 year in the current follow-up study remained nearly comparable to that at 3 months after surgery. Specifically, the eGFR decreased 2.4%, from 89.0% at 3 months to 86.9% at 12 months after surgery. Considering the spontaneous eGFR decline of 0.89–1.49 ml/min/1.73 m^2^/year associated with aging, the impact of surgery on the eGFR decline during this period appears to be mild [17–20]. Understandably, an eGFR preservation less than 90% at 1 month and 3 months was associated with eGFR preservation less than 90% at 12 months after surgery. Several groups reported predictive factors of decline in renal function after partial nephrectomy, including patient age, preoperative eGFR, tumor size and depth, warm ischemia time, and BMI [21–23]. In the current study, we also identified tumor size as a predictor for postoperative renal function decline.

The advantages of the off-clamp procedure for long-term renal function remain a topic of debate. Over a short period, no clear advantages of the off-clamp surgery were observed compared to on-clamp surgery in the robot-assisted setting [5, 24]. One meta-analysis indicated no difference in the eGFR preservation between both procedures within 6 months. However, it is essential to consider the selection bias as tumor size was significantly smaller in the off-clamp group [25]. Conversely, another meta-analysis demonstrated less decline in both short- and long-term (6 months) renal function in the off-clamp group [26]. Furthermore, off-clamp surgery was associated with a 7.3-fold decreased risk of developing stage ≥3b chronic kidney disease during a follow-up period of 2–8 years after surgery [27].

Regarding renorrhaphy, omitting cortical renorrhaphy led to eGFR preservation during the early postoperative period [7, 8, 28]. Renorrhaphy may damage the intraparenchymal vessels or cause renal artery pseudoaneurysms, both of which contribute to a reduction in vascularized renal volume. In a propensity score-matched analysis of open partial nephrectomy, renal artery pseudoaneurysms tended to be more frequent in the renorrhaphy group [9]. These findings underscore the importance of considering surgical techniques and their impact on long-term renal function when managing renal tumors.

Hemostasis using soft coagulation may also negatively impact renal function. In our previous study, the estimated blood loss served as a predictor for perioperative decline in renal function within the first three months after surgery. We hypothesized that intense use of soft coagulation to control bleeding might damage the renal parenchyma, leading to the loss of renal function [12]. Over the long term, estimated blood loss remained a predictor of renal function. However, tumor size and low eGFR preservation at 1 month emerged as notable independent predictors of renal function deterioration of renal function from 1 to 12 months. Although compensation by the contralateral kidney cannot be ignored one year after surgery, the broad use of soft coagulation may cause persistent damage to the renal parenchyma. Further studies, including assessment of divided kidney function, may clarify the effect of soft coagulation on the affected kidney.

The retrospective nature of this study and its relatively small number size are limitations of this study. In the current follow-up study, only a mild decline in eGFR was observed between three and 12 months after open partial nephrectomy. Tumor size and eGFR preservation at one month could be predictors of long-term renal function deterioration.

## Supporting information

S1 Appendix Original data. (XLSX)

## References

[1] Ficarra V, Rossanese M, Gnech M, Novara G, Mottrie A. Outcomes and limitations of laparoscopic and robotic partial nephrectomy. Curr Opin Urol. 2014;24(5):441–7. doi: 10.1097/MOU.0000000000000095 25022492

[2] Nakamura M, Ambe Y, Teshima T, Shirakawa N, Inatsu H, Amakawa R, et al. Assessment of surgical outcomes of off-clamp open partial nephrectomy without renorrhaphy for ≥T1b renal tumours. Int J Clin Oncol. 2021. 10.1007/s10147-021-01966-0 34136964

[3] Zargar H, Allaf ME, Bhayani S, Stifelman M, Rogers C, Ball MW, et al. Trifecta and optimal perioperative outcomes of robotic and laparoscopic partial nephrectomy in surgical treatment of small renal masses: a multi-institutional study. BJU Int. 2015;116(3):407–14. doi: 10.1111/bju.12933 25220543

[4] Alrishan Alzouebi I, Williams A, Thiagarjan NR, Kumar M. Omitting Cortical Renorrhaphy in Robot-Assisted Partial Nephrectomy: Is it Safe? A Single Center Large Case Series. J Endourol. 2020;34(8):840–6. doi: 10.1089/end.2020.0121 32316759

[5] Anderson BG, Potretzke AM, Du K, Vetter JM, Bergeron K, Paradis AG, et al. Comparing Off-clamp and On-clamp Robot-assisted Partial Nephrectomy: A Prospective Randomized Trial. Urology. 2019;126:102–9. doi: 10.1016/j.urology.2018.11.053 30659901

[6] Bahler CD, Cary KC, Garg S, DeRoo EM, Tabib CH, Kansal JK, et al. Differentiating reconstructive techniques in partial nephrectomy: a propensity score analysis. Can J Urol. 2015;22(3):7788–96. 26068626

[7] Bahler CD, Sundaram CP. Effect of Renal Reconstruction on Renal Function After Partial Nephrectomy. J Endourol. 2016;30 Suppl 1:S37-41. 10.1089/end.2016.0055 26864480

[8] Bertolo R, Campi R, Mir MC, Klatte T, Kriegmair MC, Salagierski M, et al. Systematic Review and Pooled Analysis of the Impact of Renorrhaphy Techniques on Renal Functional Outcome After Partial Nephrectomy. Eur Urol Oncol. 2019;2(5):572–5. doi: 10.1016/j.euo.2018.11.008 31412012

[9] Tachibana H, Takagi T, Kondo T, Ishida H, Tanabe K. Comparison of perioperative outcomes with or without renorrhaphy during open partial nephrectomy: A propensity score-matched analysis. Int Braz J Urol. 2018;44(3):467–74. doi: 10.1590/S1677-5538.IBJU.2016.0581 29244272 PMC5996815

[10] Furukawa J, Miyake H, Hinata N, Muramaki M, Tanaka K, Fujisawa M. Renal Functional and Perioperative Outcomes of Selective Versus Complete Renal Arterial Clamping During Robot-Assisted Partial Nephrectomy: Early Single-Center Experience With 39 Cases. Surg Innov. 2016;23(3):242–8. doi: 10.1177/1553350615610648 26459499

[11] Mattevi D, Luciani LG, Mantovani W, Cai T, Chiodini S, Vattovani V, et al. Fluorescence-guided selective arterial clamping during RAPN provides better early functional outcomes based on renal scan compared to standard clamping. J Robot Surg. 2019;13(3):391–6. doi: 10.1007/s11701-018-0862-x 30094595

[12] Nakamura M, Kameyama S, Ambe Y, Teshima T, Izumi T, Tsuru I, et al. Predictive factors for postoperative renal function after off-clamp, non-renorrhaphy partial nephrectomy. Transl Androl Urol. 2022;11(9):1226–33. doi: 10.21037/tau-22-321 36217403 PMC9547154

[13] Hongo F, Kawauchi A, Ueda T, Fujihara-Iwata A, Nakamura T, Naya Y, et al. Laparoscopic off-clamp partial nephrectomy using soft coagulation. Int J Urol. 2015;22(8):731–4. doi: 10.1111/iju.12808 25989004

[14] Nakamura K, Imamura Y, Yamamoto S, Sazuka T, Sakamoto S, Ichikawa T. Soft coagulation in robot-assisted partial nephrectomy without renorrhaphy: Comparison with standard suture. Int J Urol. 2020;27(4):352–4. doi: 10.1111/iju.14195 32036616

[15] Ooi A. Advances in hereditary leiomyomatosis and renal cell carcinoma (HLRCC) research. Semin Cancer Biol. 2020;61:158–66. doi: 10.1016/j.semcancer.2019.10.016 31689495 PMC7078051

[16] Kutikov A, Uzzo RG. The R.E.N.A.L. nephrometry score: a comprehensive standardized system for quantitating renal tumor size, location and depth. J Urol. 2009;182(3):844–53. doi: 10.1016/j.juro.2009.05.035 19616235

[17] Pottel H, Delanaye P, Weekers L, Selistre L, Goffin K, Gheysens O, et al. Age-dependent reference intervals for estimated and measured glomerular filtration rate. Clin Kidney J. 2017;10(4):545–51. doi: 10.1093/ckj/sfx026 28852494 PMC5570001

[18] Poggio ED, Rule AD, Tanchanco R, Arrigain S, Butler RS, Srinivas T, et al. Demographic and clinical characteristics associated with glomerular filtration rates in living kidney donors. Kidney Int. 2009;75(10):1079–87. doi: 10.1038/ki.2009.11 19212414 PMC2713659

[19] Noronha IL, Santa-Catharina GP, Andrade L, Coelho VA, Jacob-Filho W, Elias RM. Glomerular filtration in the aging population. Front Med (Lausanne). 2022;9:769329. doi: 10.3389/fmed.2022.769329 36186775 PMC9519889

[20] Pottel H, Hoste L, Yayo E, Delanaye P. Glomerular Filtration Rate in Healthy Living Potential Kidney Donors: A Meta-Analysis Supporting the Construction of the Full Age Spectrum Equation. Nephron. 2017;135(2):105–19. doi: 10.1159/000450893 27764827

[21] Yu YD, Nguyen NH, Ryu HY, Hong SK, Byun SS, Lee S. Predictors of renal function after open and robot-assisted partial nephrectomy: A propensity score-matched study. Int J Urol. 2019;26(3):377–84. doi: 10.1111/iju.13879 30582218

[22] Takagi T, Kondo T, Iizuka J, Tomita E, Kobayashi H, Hashimoto Y, et al. Predictors for postoperative renal function after open partial nephrectomy: including postoperative biomarkers. Int J Urol. 2012;19(9):823–8. doi: 10.1111/j.1442-2042.2012.03037.x 22568789

[23] Chan AA, Wood CG, Caicedo J, Munsell MF, Matin SF. Predictors of unilateral renal function after open and laparoscopic partial nephrectomy. Urology. 2010;75(2):295–302. doi: 10.1016/j.urology.2009.09.027 19963245

[24] Anderson BG, Potretzke AM, Du K, Vetter J, Figenshau RS. Off-clamp robot-assisted partial nephrectomy does not benefit short-term renal function: a matched cohort analysis. J Robot Surg. 2018;12(3):401–7. doi: 10.1007/s11701-017-0745-6 28861728

[25] Antonelli A, Veccia A, Francavilla S, Bertolo R, Bove P, Hampton LJ, et al. On-clamp versus off-clamp robotic partial nephrectomy: A systematic review and meta-analysis. Urologia. 2019;86(2):52–62. doi: 10.1177/0391560319847847 31179885

[26] Deng W, Liu X, Hu J, Chen L, Fu B. Off-clamp partial nephrectomy has a positive impact on short- and long-term renal function: a systematic review and meta-analysis. BMC Nephrol. 2018;19(1):188. doi: 10.1186/s12882-018-0993-3 30064370 PMC6069776

[27] Simone G, Capitanio U, Tuderti G, Presicce F, Leonardo C, Ferriero M, et al. On-clamp versus off-clamp partial nephrectomy: Propensity score-matched comparison of long-term functional outcomes. Int J Urol. 2019;26(10):985–91. doi: 10.1111/iju.14079 31342589

[28] Bahler CD, Dube HT, Flynn KJ, Garg S, Monn MF, Gutwein LG, et al. Feasibility of omitting cortical renorrhaphy during robot-assisted partial nephrectomy: a matched analysis. J Endourol. 2015;29(5):548–55. doi: 10.1089/end.2014.0763 25616087

